# Novel approach to enhance coastal habitat and biotope mapping with drone aerial imagery analysis

**DOI:** 10.1038/s41598-020-80612-7

**Published:** 2021-01-12

**Authors:** João Gama Monteiro, Jesús L. Jiménez, Francesca Gizzi, Petr Přikryl, Jonathan S. Lefcheck, Ricardo S. Santos, João Canning-Clode

**Affiliations:** 1 MARE - Marine and Environmental Sciences Centre, ARDITI - Agência Regional para o Desenvolvimento da Investigação, Tecnologia e Inovação, Funchal, Madeira Portugal; 2 Department of Biology and Ecology, Faculty of Science, University of Ostrava, Ostrava, Czech Republic; 3 Tennenbaum Marine Observatories Network, MarineGEO, Smithsonian Environmental Research Center, Edgewater, MD USA; 4 Ministry of the Sea, Avenida Doutor Alfredo Magalhães Ramalho, 1465-165 Algés, Portugal; 5 University of the Azores, Horta, Portugal; 6 Smithsonian Environmental Research Center, Edgewater, MD USA

**Keywords:** Biodiversity, Biooceanography, Ecological modelling, Marine biology, Ecosystem ecology, Image processing, Machine learning

## Abstract

Understanding the complex factors and mechanisms driving the functioning of coastal ecosystems is vital towards assessing how organisms, ecosystems, and ultimately human populations will cope with the ecological consequences of natural and anthropogenic impacts. Towards this goal, coastal monitoring programs and studies must deliver information on a range of variables and factors, from taxonomic/functional diversity and spatial distribution of habitats, to anthropogenic stress indicators such as land use, fisheries use, and pollution. Effective monitoring programs must therefore integrate observations from different sources and spatial scales to provide a comprehensive view to managers. Here we explore integrating aerial surveys from a low-cost Remotely Piloted Aircraft System (RPAS) with concurrent underwater surveys to deliver a novel approach to coastal monitoring. We: (i) map depth and substrate of shallow rocky habitats, and; (ii) classify the major biotopes associated with these environmental axes; and (iii) combine data from i and ii to assess the likely distribution of common sessile organismal assemblages over the survey area. Finally, we propose a general workflow that can be adapted to different needs and aerial platforms, which can be used as blueprints for further integration of remote-sensing with in situ surveys to produce spatially-explicit biotope maps.

## Introduction

Coastal habitats and the biodiversity they support have been declining at unprecedented rates, and with them critical ecosystem services that support human well-being and livelihoods^1–4^. Understanding the spatial distribution and function of different coastal habitats, the biodiversity and community structure, and their susceptibility to disturbances is essential towards effectively managing human impacts and ensuring the reliable provision of economically-valuable resources^4–7^. Under the EU Marine Strategy Framework Directive (2008/56/EC), for example, national monitoring programs of European Union Member States are expected to deliver information on a variety of variables from species diversity and habitat distribution, to anthropogenic stress indicators, such as land use, fisheries and pollution.

Acquiring information on coastal and shallow submerged habitats and resident biota has traditionally relied on Underwater Visual Census (UVC) and SCUBA diving^8,9^. Over the last decades, developments in scientific diving and underwater photography and video have increasingly enhanced the speed and accuracy of underwater data acquisition, annotation and analysis^7,9–14^. Despite such advances, there are still several constraints and limitations associated with underwater surveys by scuba divers: in addition to requiring high-level of expertise, SCUBA diving and UVC have limited bottom times and sampling areas, producing (geographically) discrete spatial data points. Additional challenges include difficulties in producing or extending high-resolution spatially explicit information in a highly dynamic, three-dimensional environment and mapping physiographic traits such as bathymetry and bottom nature. Most bathymetry assessments and maps rely on surveys with acoustic sensors (single-, multi-beam or side-scan sonar), which are generally costly, time consuming, require high-level of post processing expertise and have numerous operational constrains in nearshore areas^6,8,15^, often leading to the adoption of optical derived bathymetry and other remote sensing methods.

In recent years, satellite remote sensing technology has yielded considerable success in developing optical analysis approaches to derive bathymetry and to map near-shore environments such as coral reefs, marshes and mangroves^15–19^. However, acquiring satellite imagery with higher spatial resolution (e.g. < 1 m) than the publicly available databases (e.g. Sentinel-2 at 10 m) and surveys with active sensor Light Detection and Ranging (LiDAR) can be prohibitively costly^8,20^. In addition, outputs from these approaches typically provide physiographic information and general traits to classify different habitat types (e.g. rocky reef, sand, fore- and back-reef), but usually do not integrate information on how different biological assemblages are distributed over the mapped physiographic habitats^17,21–23^.

The recent development of inexpensive commercial off-the-shelf (COTS) drones and other advanced Remotely Piloted Aircraft Systems (RPAS) has made high-tech aerial imagery platforms easily and widely accessible. Automated flight ability at low altitudes enables RPAS to produce aerial imagery with higher resolution than that achieved by current satellites or by manned aerial platforms. Despite a broad range of terrestrial applications (from agriculture and architecture to wildlife monitoring and conservation^24,25^), RPAS have only recently started to be employed in the marine realm. Nevertheless, they have already demonstrated a variety of applications, including assessment of intertidal areas, shallow coral reefs and estuarine algal cover, the detection of fish nursery habitats and marine litter and the production of bathymetry maps^20,26–31^. Despite these advances, the studies have mostly been focused in delivering information on a key trait or feature (e.g. depth, algal cover), but have yet to combine and integrate such remote sensed data with biological data from underwater surveys enabling the extrapolation of biological communities’ distribution across physiographic habitats or varying conditions.

Tackling the trade-offs among methods and techniques to map and describe coastal habitats is a key challenge for monitoring programs to provide maximally useful information for marine spatial planning and management^6,32^. To that purpose, the present study explores the integration of scientific diving and underwater surveys with aerial surveys using a low-cost recreational RPAS to map and provide information on the distribution of sessile biotopes: distinct assemblages of sessile organisms occurring under specific environmental conditions. We used underwater photoquadrat imagery to assess sessile benthic assemblages and RPAS based aerial surveys to construct a georeferenced ortho-photomosaic and a Digital Surface Model (DSM) of a small bay in Madeira Island (NE Atlantic). We then used multivariate statistical routines, aerial imagery photogrammetry, Object Based Imagery Analysis (OBIA) and automated classification to identify and provide information on the presence and distribution of distinct biotopes over different depth-substrate classes within the study area. We further discuss the limitations, benefits and possible improvements of such an approach, and propose some general guidelines for leveraging this strategy towards mapping local coastal habitats and associated biotopes.

## Results

### Mapping coastal habitat physiography from the sky

**Figure 1:**
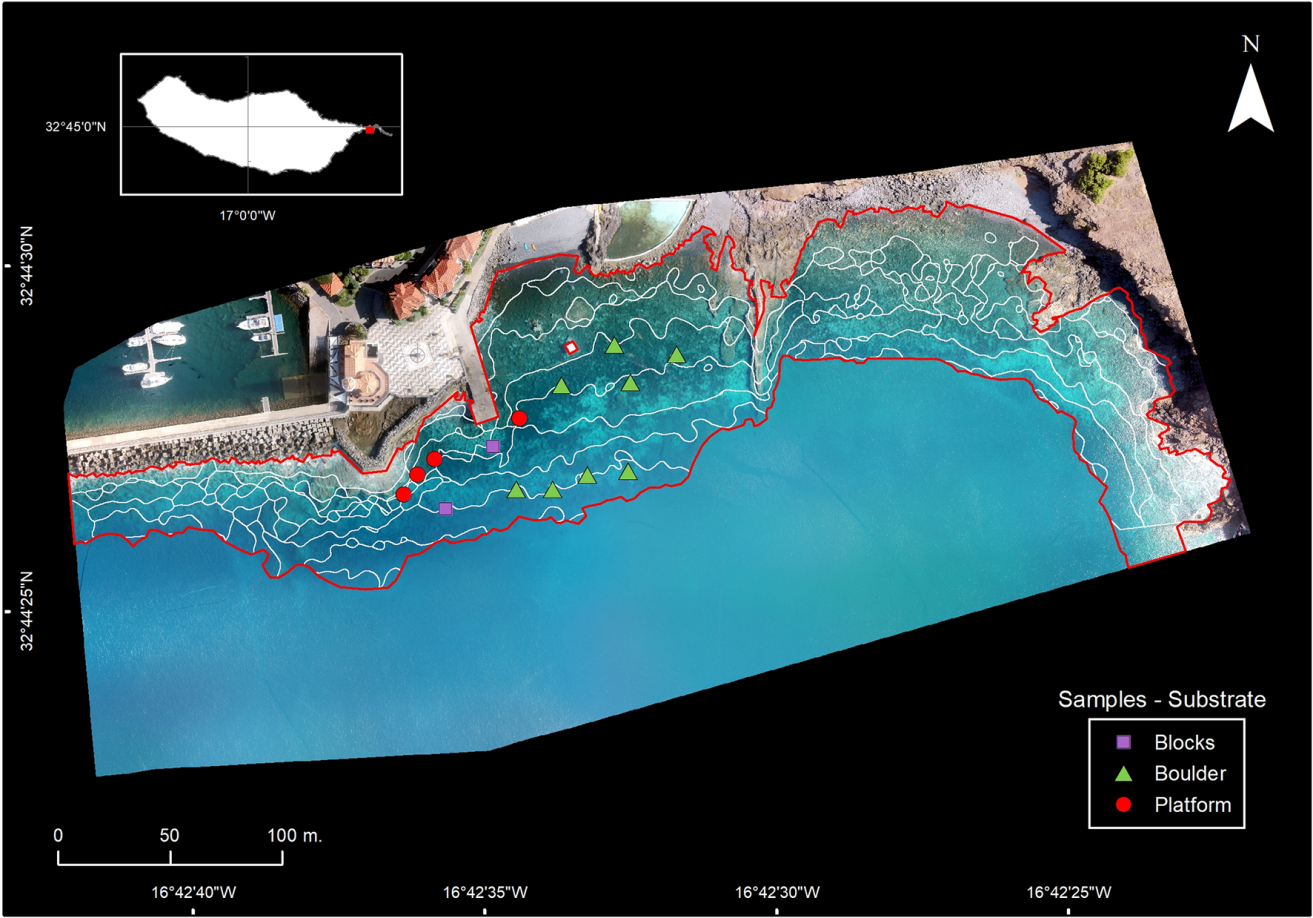
Ortho-photo mosaic of study area in Madeira island (location on top-left box) with discrete boundaries for depth classes (white contours) over assessment target area (red-contour) and location of underwater surveys (shape and colour indicate dominant substrate; bottom-right label box). Map and figure generated in ESRI ArcGIS Desktop v10.3.1.

Three aerial survey flights collected 213 images and allowed the construction of an ortho-photomosaic and a DSM^33^ covering over 10 ha of a sheltered bay, in the southeastern section of Madeira Island (Fig. 1, Supplementary Fig. S1), where shallow areas (< 13 m depth) are mostly comprised of rocky bottoms. Combining data on depth and substrate type collected during underwater surveys (Fig. 1) with a visual inspection of high-resolution aerial imagery, enabled to select a training set for supervised classifications of a target area of interest using eCognition Essentials (v1.3).

**Figure 2:**
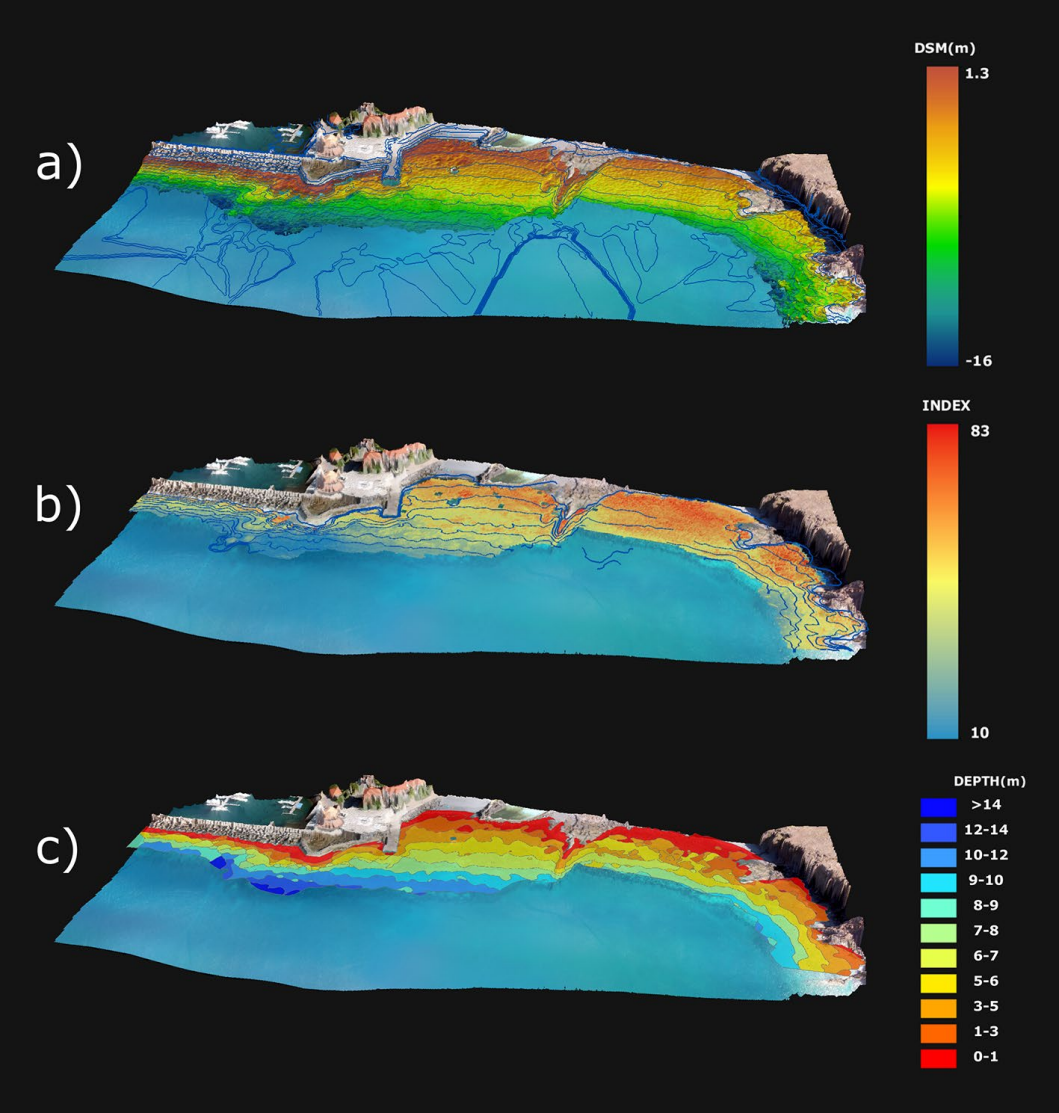
Optically Derived Bathymetry estimation from aerial imagery mosaic and photogrammetric generated digital surface model (DSM); with: (**a**) DSM depth variation (m) over rocky bottom and DSM contours with 2-m interval (blue lines); (**b**) combined (normalized) ln-RED index and ratio of ln-Green to ln-Blue index and smoothed, contextually edited, depth contours used for Optically Derived Bathymetry estimation using OBIA (see above), and; (**c**) resulting estimated depth classes. Map and figure generated in ESRI ArcGIS Desktop v10.3.1.

**Figure 3:**
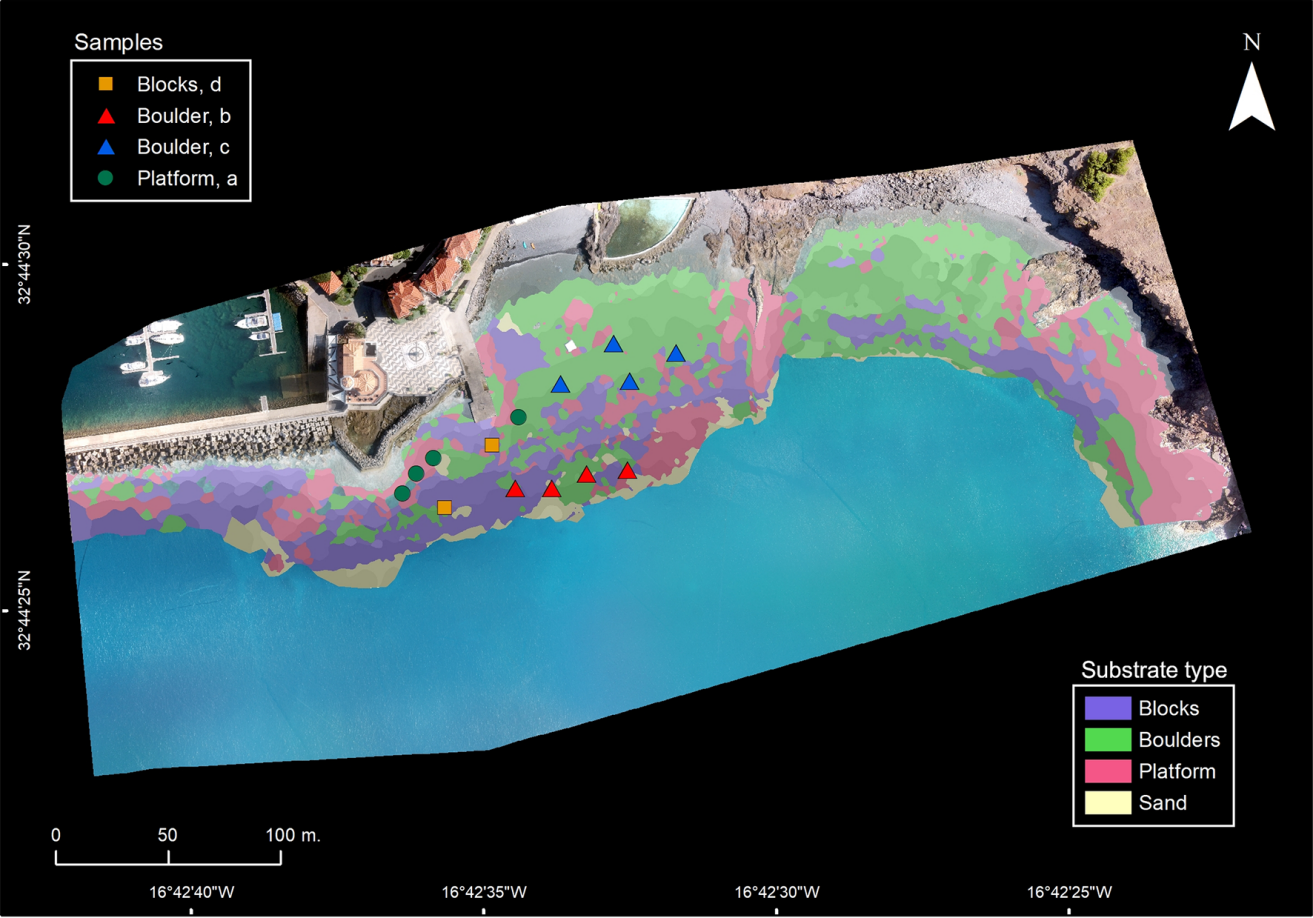
Substrate map derived from ortho-photomosaic analysis (coloured layers indicate substrate type; bottom-right label box) with location of underwater photoquadrat surveys, (shape indicates dominant substrate; colour indicates organism assemblage; top-left box). Map and figure generated in ESRI ArcGIS Desktop v10.3.1.

Based on the Digital Surface Model (DSM), on the reflectance index maps (Fig. 2a, b) and on the photo-mosaic generated with Pix4D Mapper (average Ground Sampling Distance, GSD = 5.65 cm/pixel)^33^, we produced a baseline bathymetry map (Fig. 2c) and an optically derived bathymetry over rocky substrate, with 11 discrete depth classes (each ranging between 1 and 3 m) from the surface to 14 m depth (Figs. 1, 2c). Segmentation and substrate classification of individual discrete depth classes assured a low variation in reflectance due to depth-related attenuation of different wavebands^17,23,34,35^, ensuring the classification process was focused on substrate-related differences in reflectance. Merging information from all depth classes resulted in a map of the target area containing information with discrete depth classes and boundaries for three major categories of rocky substrate: "boulders" (mostly with rocks between 15 and 100 cm in diameter), "blocks" (rocks with 100 cm diameter or more) or "platforms" (horizontal or near horizontal rocky “plateau" (Fig. 3).

### Identifying biotopes

The analysis of the photoquadrat samples (n = 14 sets of six photo-quadrats) identified four unique biotic assemblages (labelled from a. to d.) based on Bray–Curtis Similarities between samples and a SIMilarity PROFile routine (SIMPROF)^36,37^. A SIMilarity PERcentage analysis (SIMPER) revealed individual taxa average abundance, similarity within each assemblage and contribution to ordination into the four assemblages (Table 1). These distinct organism assemblages were further corroborated by an ANalysis Of SIMilarities (ANOSIM) of the individual quadrats (R:0.637; p:0.01), revealing that the SIMPROF grouping is also significant when looking into similarities between each individual quadrat.

**Table 1:** Taxa composition and contribution to SIMPROF generated organism assemblages (OA) present at 3–6 m and 9–11 m depth over rocky bottom: Average Abundance (Av. Abundance) based on Untransformed data; Average Similarity (Av. Sim.) and Individual (Ind. Cont.) and Cumulative (Cum. Cont.) Taxa Contribution (%), based on Square rooted transformed data.

OA	Taxa	Untransformed	Sq. root transformed		
		Av.Abundance (%)	Av.Sim. (%)	Ind.Cont. (%)	Cum.Cont. (%)
a	Turf	41.11	26.88	34.02	34.02
	*Asparagopsis* sp.	33.14	20.7	26.2	60.22
	Biofilm w/ silt	11.88	11.11	14.05	74.28
	*Dictyota* sp.	7.63	10.22	12.93	87.21
	CCA	5.13	8.73	11.05	98.26
	*Spongionella* sp.	0.2	0.36	0.46	98.72
	*Reptadeonella* sp.	0.16	0.35	0.44	99.16
	*Crambe* sp.	0.11	0.34	0.43	99.58
	*Macrorhynchia* sp.	0.33	0.33	0.42	100
b	Biofilm w/ silt	48.68	28.37	34.53	34.53
	Turf	19.01	16.84	20.5	55.03
	*Macrorhynchia* sp.	13.04	14.03	17.08	72.11
	CCA	9	9.19	11.18	83.29
	*Lobophora* sp.	3.4	5.44	6.62	89.91
	*Asparagopsis* sp.	4.14	3.74	4.55	94.47
	*Dictyota* sp.	1.86	3.74	4.55	99.01
	Rodolith	0.44	0.5	0.61	99.63
	*Crambe* sp.	0.11	0.31	0.37	100
c	Biofilm w/ silt	38.23	25.38	34.08	34.08
	Turf	38.37	23.85	32.03	66.11
	CCA	7.94	8.65	11.61	77.72
	*Dictyota* sp.	2.99	5.68	7.63	85.35
	*Asparagopsis* sp.	4.41	4.74	6.37	91.71
	*Halopteris* sp.	5.1	3	4.03	95.75
	*Ircinia* sp.	2.67	2.83	3.81	99.55
	*Crambe* sp.	0.11	0.33	0.45	100
d	CCA	45.6	27.97	63.94	63.94
	Biofilm w/ silt	23.68	13.69	31.29	95.23
	Turf	0.47	2.09	4.77	100

**Figure 4:**
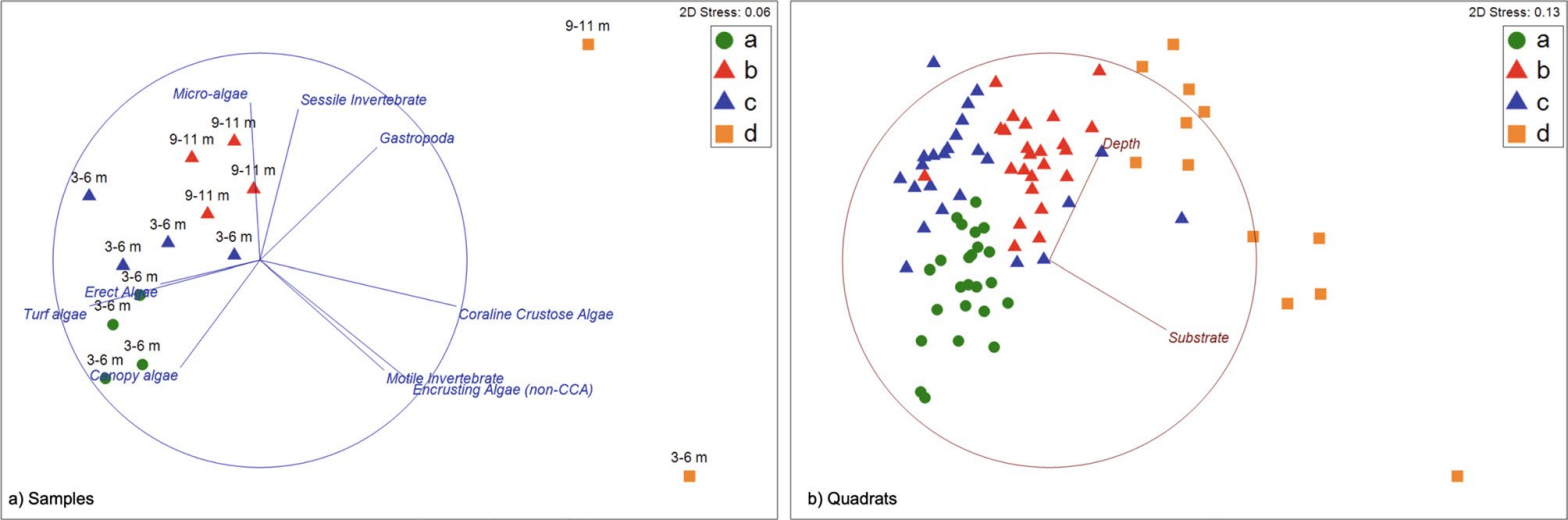
Non-metric Multidimensional Scaling plots of Bray–Curtis similarity ordinations of samples and (left) and quadrats (right) labelled by organism assemblage (top right corner); (**a**) Photoquadrat Survey Samples (n = 14) with correlation vectors of major taxonomic groups (overlay in blue lines), and; (**b**) Individual Quadrats (n = 84) with correlation vectors of normalised variation in Depth and Substrate categories groups (overlay in red lines). Figure generated in PRIMER v7 (with PERMANOVA add-on).

**Figure 5:**
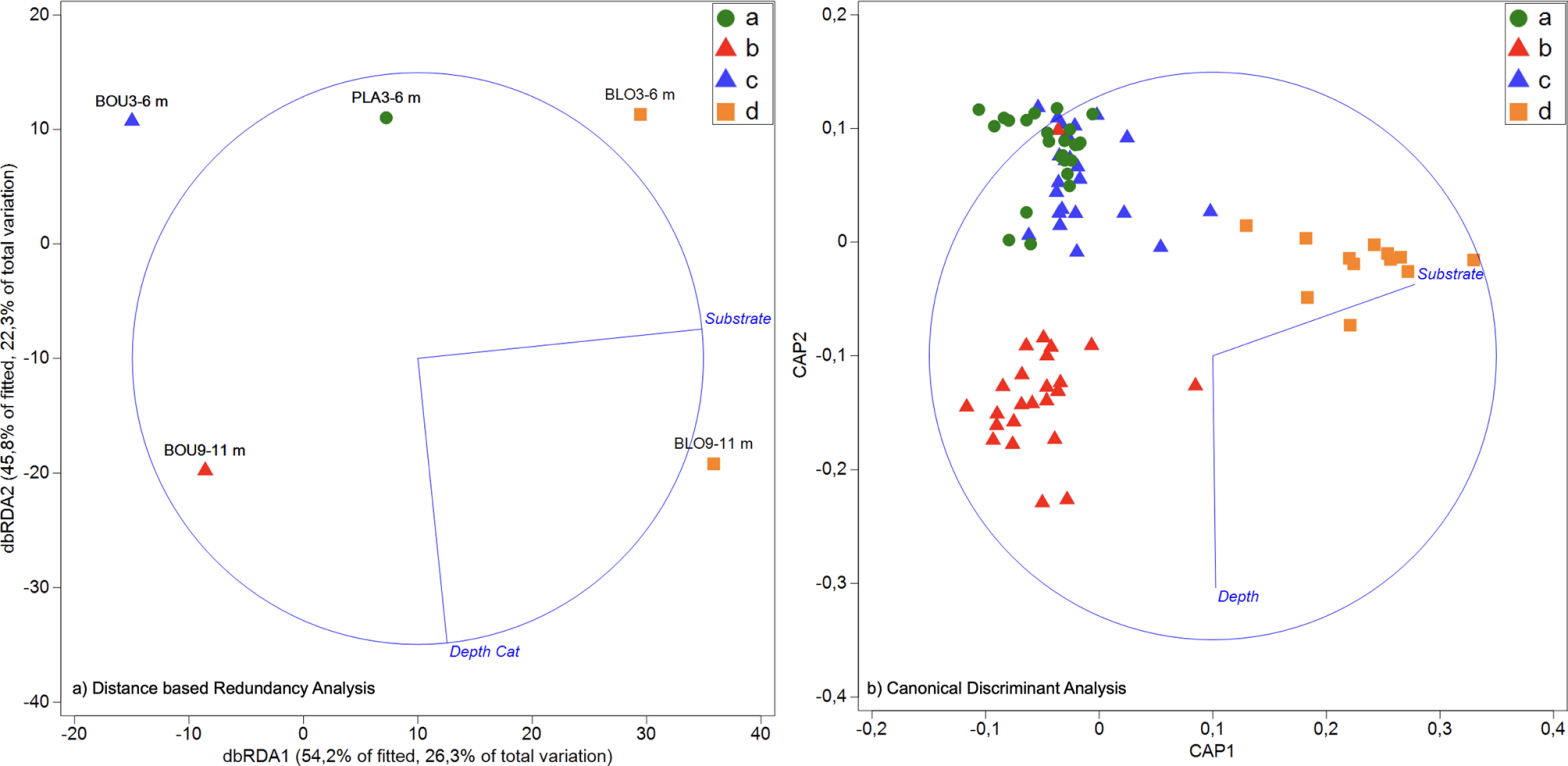
Predicting assemblages (**a**–**d**) through DistLM and correlations of Depth and Substrate (blue vectors overlay) with ordinations; (**a**) correlations with dbRDA axes most explaining total variation in samples, illustrating best solution from a Distance based Linear Model (left), and; (**b**) correlations with Canonical axes (CAP1 and CAP2) best discriminating sample-derived SIMPROF grouping of individual quadrats (right). Figure generated in PRIMER v7 (with PERMANOVA add-on).

A visual inspection of a non-metric Multidimensional Scaling plots labelled with SIMPROF-generated organism assemblages (Fig. 4) revealed: (i) clear grouping of samples and quadrats; (ii) how major taxonomic groups varied between assemblages, and; (iii) a strong correlation between surveyed substrates and depth classes with identified biotic assemblages (Supplementary Table S1). Indeed, a Distance-Based Linear Model using Depth and Substrate categories (normalised) as predictor variables indicated that both were significant in shaping sample ordination (Fig. 5; Supplementary Table S2), with the best overall solution including both predictors and explaining 48% of the variation in sample ordination (and more than 32% of the variation when considering the individual quadrats). Having established that individual quadrats can be significantly grouped into the four assemblages (ANOSIM, R:0.637; p:0.01), we used Canonical Discriminant Analysis plot (CDA) to further illustrate how depth and substrate best correlate with quadrat grouping into the four organism assemblages (Fig. 5).

The matching and correlations between biological assemblages and substrate and depth conditions allowed the identification of four conspicuous biotopes^38^: Platforms at 4–6 m dominated by *Turf* and the canopy algae *Asparagopsis* sp. (Assemblage a.); Boulders at 9–11 m dominated by *Biofilm with silt, Turf* and the hydroid *Macrorhynchia* sp. (Assemblage b.); Boulders at 4-–6 m also dominated by *Biofilm with silt* and *Turf* but with *Coralline Crustose Algae (CCA*) being the third most abundant category and with some taxa being absent (Assemblage c.), and lastly; Blocks at 4–6 m and at 9–11 m with *CCA* being the most abundant category and dominating this substrate along with *Biofilm with silt* (Assemblage d.)*.*

Overall, photoquadrat survey data and SIMPER analysis also revealed that the most common categories within the whole study site were *Biofilm with silt* (i.e. a microalgal and hydroid assemblage partially covered with silt) and *Turf* (i.e. a mixed algal assemblage with < 3 cm, mostly composed by small filamentous algae including species such as species such as *Ceramium* sp., *Polysiphonia* sp. and *Jania* sp.), whereas erect and/or canopy forming algae were generally lacking and coralline crustose algae (CCA) were especially abundant over large blocks (Table 1).

### Habitat mapping and biotope distribution

**Figure 6:**
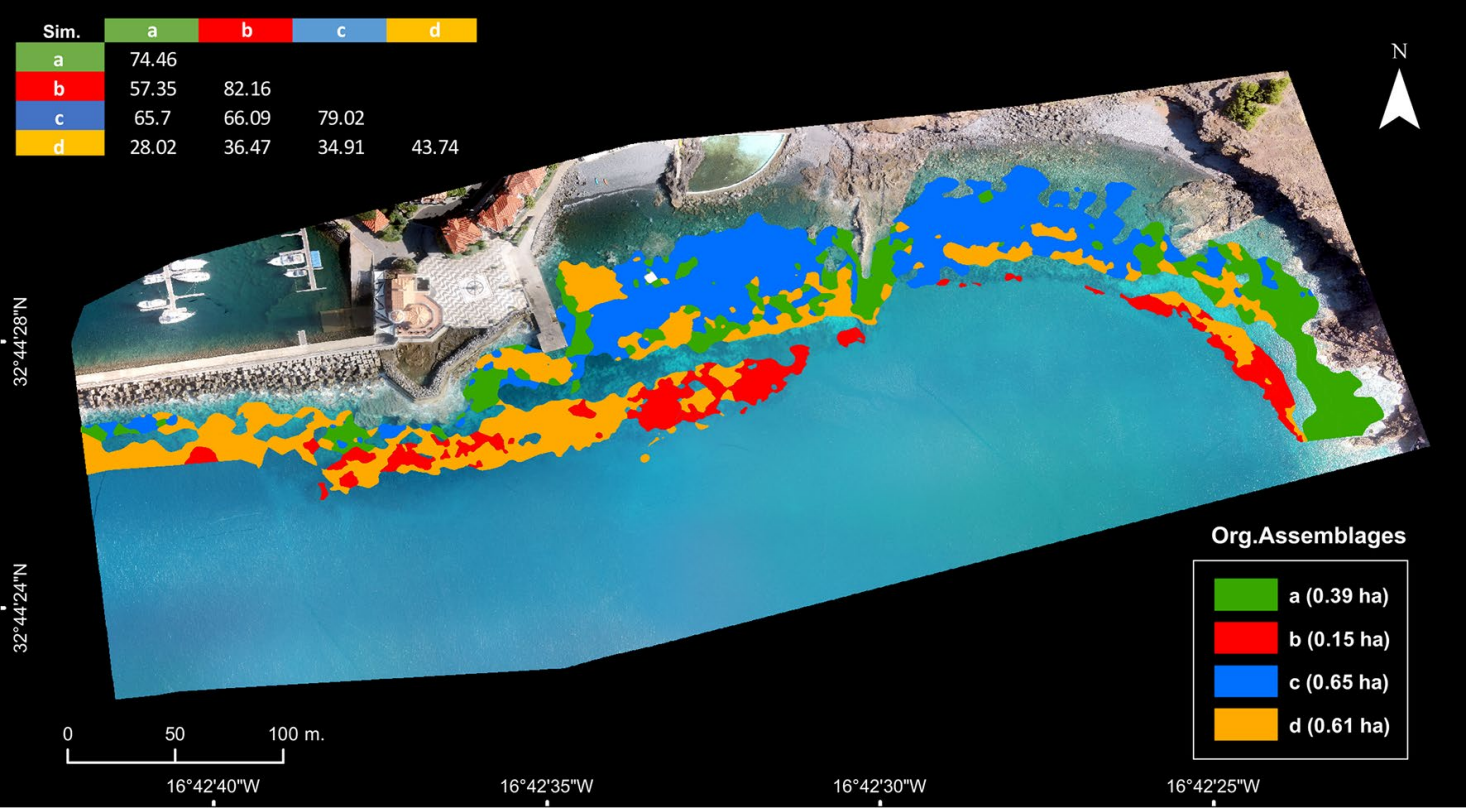
Extrapolated biotope (**a**–**d**) distribution (coloured map) and estimated area of unique organism assemblages (lower right corner) over distinct rocky substrate categories within the surveyed depth classes (biotope similarity matrix between biotopes on top-left corner). Map and figure generated in ESRI ArcGIS Desktop v10.3.1.

Assuming biotopes as areas with uniform environmental conditions where particular organism assemblages occur^38^, we combined information on habitat physiography (“Mapping coastal habitat physiography from the sky” section) and conspicuous organism assemblages (“Identifying biotopes” section) to identify biotopes and extrapolate their spatial distribution based on matching conditions and correlations (Fig. 5; Supplementary Table 1). Achieved by selecting merged segments of particular physiographic conditions and label them with matching biotope, the extrapolated distribution (Fig. 6) was then validated by analysing the similarity (ANOSIM) of independent, georeferenced, transect-based benthic survey data (n = 8, 10 m transects with 100-point intersections each). Very high congruence was found between the observed data ordination and the predicted biotope grouping (R:0.92; p:0.02).

This extrapolative approach produced a biotope map with the distribution of four distinct benthic assemblages (Fig. 6) over 53% of the rocky bottoms (1.8 ha of a total of 3.37 ha of rocky bottoms). Remaining rocky bottoms were outside of the target depth classes (i.e. 4–6 m and 9–11 m). Within the mapped area, Boulders at 4–6 m dominated by *Biofilm with silt* and *Turf* (Assemblage c.) is the most common biotope, covering 0.65 ha. Inspecting estimated areas and relative cover (Table 1; Supplementary Table S1), reveals that biotopes where boulders are dominated by *Biofilm with silt* and *Turf* (Assemblages b. and c.) cover up to 44% of the assessed area. Large Blocks harbouring the least diverse and structurally complex assemblage (Assemblage d.) is estimated to be the second most common biotope in the area (34%). Platforms at 4–6 m with the most structurally complex assemblage (Assemblage a.) comes in third, with an estimated cover of 22% of the assessed area. This spatially explicit data is key to understanding how fauna and flora are distributed, how common different biotopes are, and to effectively strategize for marine spatial planning and management.

## Discussion

**Figure 7:**
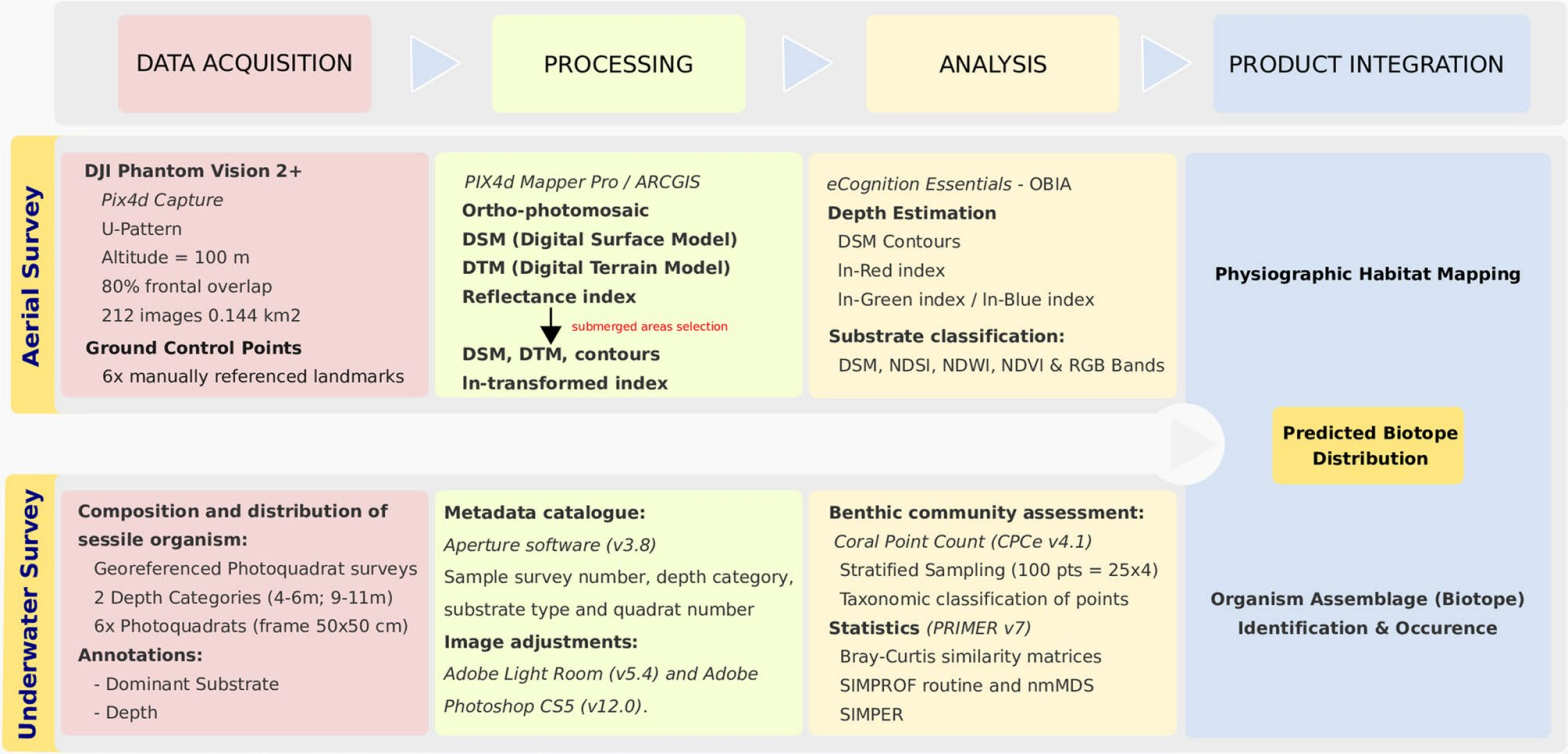
Workflow blueprint for implementing a coastal habitat mapping novel workflow integrating aerial RPAS based survey and underwater photo-based benthos surveys to identify and predict biotope distribution.

Despite all the technological and methodological advances in marine science during the last decades, ecology studies focusing in shallow marine habitats still overwhelmingly rely on manned underwater surveys to collect samples, assess ecological traits and examine biological responses^7,9–14^. Typically more accessible and with more direct pressure from humans, coastal shallow waters have been the target of extensive research and the stage for numerous studies, however, the ability to assess, produce maps or predict distribution of conspicuous organism assemblages residing within specific environmental conditions in coastal waters is still challenging and often limited^6,8,39^. In this work, we propose a strategy (Fig. 7) that extends the utility of underwater survey data by integrating it with remotely sensed imagery and multivariate statistics to generate maps of benthic assemblages at a small spatial scale (i.e. sub-meter). The strategy relies on well-established methodological principles and statistical approaches^17,34–37,40^, but combines them in a unique and novel fashion to assess key physiographic features from low-altitude aerial imagery and employ it to extrapolate the distribution of distinct biotopes to map them over a target survey area.

Previous studies have already explored the use of remote sensing from satellite and RPAS aerial imagery for shallow-water marine habitat mapping and assessments^8,16,17,20,26,28,40–42^, but they have thus far been limited in integrating data from biological communities, and have mostly included image classification to identify broad habitats categories based on reflectance and spectral profiles without assessing or describing the associated community. Recently, RPAS surveys have also been used to map coastal topographical features, to assess intertidal communities and shallow corals^28,31,43,44^, but there are not many cases of RPAS remote sensing being employed to map or model the distribution biological communities in shallow submerged habitats.

Despite the efforts to enhance the ability for aerial imagery to “penetrate” through water^26^ it is still not possible to use them to discriminate submerged communities. Until this becomes a reality, we provide here an alternate approach to leverage existing in situ data on community composition with physiographic maps generated from RPAS imagery to statistically identify biotopes and determine their distribution. Our approach provides several advantages over remote sensing alone: (i) additional detailed information on biota distribution, community structure and composition; (ii) an unbiased method towards identifying statistically different organism assemblages with no a priori determination, and; (iii) a path towards classifying biotope and organism assemblages distribution, based on their correlation with assessed and mapped environmental conditions.

To that purpose, having a discrete map for substrate and depth was essential, to which we opted to rely on eCognition supervised classification using both ln-RED and the ratio of ln-GREEN to ln-BLUE with ground truth data as samples. The inclusion of the ln-RED index, which has much less penetration in water^34,35^, allowed a better detection of shallow areas (0–3 m). The original use of Object Based Imagery Analysis (OBIA) to assess depth allowed to create segments and classify it into classes (each with 1 to 3 m range), enabling the generation of 11 discrete depth classes that would sequentially be used to assess substrate based on reflectance variation^8,17^. This innovative strategy to map these physiographic habitat traits, that are of key importance for residing organisms, can be easily scaled up and/or adapted to fit other data sources (e.g. sonar surveys, existing bathymetry maps, satellite imagery) or additional spatially available variables or predictors (e.g. chlorophyll-a, pollution sources).

Despite being fairly simple to operate, multirotor RPAS similar to the one used in our approach require reasonable conditions of weather and natural light in order to enable sensing sub-surface conditions. In general, multirotor drones can operate with up to 10 knot winds, however, to assess depth and submerged substrates, it is required to have winds and ocean conditions with a maximum of 2 under the modern Beaufort scale (with no waves or with small waves where crests have a glassy appearance and do not break). The time of the day and how high the sun is in the sky is another consideration that must be taken, for reducing sun glint^20^, with ideal conditions being clear skies and the sun at a low angle. One additional limitation in using RPAS as a remote sensing platform is linked to legal constraints and authorizations required to fly in some locations (e.g. protected areas, airport vicinities, high population density areas), which need to be considered when planning RPAS flight operations and aerial surveys. Finally, like any OBIA approach, depth estimation by imagery analysis is limited by color variation over depth, which generally limits it to a maximum of 30 m depth, in very clear waters^15–17^. More importantly, as mosaic construction and DSM generation requires individual images to overlap over common features, aerial surveys over water generally require that the shore-line is in-picture, which inevitably limits (to any given altitude) the maximum distance from the shore-line that an aerial survey flight-path can be used and included in depth estimation.

In spite of such limitations, RPAS-based aerial surveys and the approach we propose provide several advantages over traditional satellite assessments. First, the resolution of the imagery obtained from RPAS is, at least currently, much higher than most publicly-available satellite imagery (i.e. < 0.1 m vs 10 m for Sentinel-2), making it potentially easier to discriminate key features that might be obscured in lower resolution imagery. Second, satellites obtain a fixed set of images every day regardless of the local conditions. Thus, certain times and locations may be disrupted by high cloud cover, high surf, sun glare, or other conditions that obscure the benthos and prevent acquisition of useful imagery beyond the target dates. In contrast, RPAS—while requiring similar conditions—are highly flexible, and can therefore be deployed when conditions are most amenable during the desired study window. Thus, RPAS produces higher resolution outputs and can acquire more relevant and targeted imagery than publicly available satellite data, while maintaining a lower cost than other aerial survey platforms and commercial, high resolution satellite data (e.g. Worldview-3 and 4).

Like many other studies relying on modelling and statistics to predict species distribution and habitat suitability, the present research and workflow has limitations inherent with the “translation” of complex natural processes and mechanisms into more or less complex models and mathematical expressions^45,46^. Modelling species distribution is often limited by the quality of the data included but also due to lack of data or mismatching spatial scales between datasets and, the present study, is no exception. The future inclusion of additional layers of environmental data and other predictors, for example, could enhance the level of detail of predicted distributions. In our particular application, biotopes are classified based on statistically-validated “groupings” rather than truly predictive models: in other words, the classification is binary (either the environmental conditions match a particular assemblage or not). Consequently, there is no quantification of error or uncertainty as one might obtain from a statistical model, although we point out the high degree of agreement between the assigned groupings and the observed biotopes in our independent validation suggests that error is, at least in our case, minimal. On the other hand, grouping organisms into four biotopes based on two axes (depth and substrate type), as we have here, is simple, intuitive and requires less data and little experience in advanced modelling techniques, key characteristics that are likely to be attractive to managers who are often seeking simple and explainable solutions to enact effective management.

The present research sets a path for future studies to build up from and for further enhancing the integration of RPAS-based aerial surveys with other data sources, namely by:

i) using RPAS platforms with additional sensor payload that can be used to detect or characterise specific features (i.e. multispectral or hyperspectral sensors for bathymetry or normalised difference vegetation index assessments^18,30,47^);
ii) standardising reflectance reads and combining optically derived bathymetry from shallow waters with sonar data (i.e. for integration of bathymetry of deeper waters) to improve shallow waters bathymetric surveys from aerial imagery;
iii) including additional information on biological and environmental conditions (e.g. biotopes with finer-scale taxonomy, temperature and turbidity, marine traffic and coastal development) can provide more detailed community structure or distribution data;
iv) including more sophisticated and complex Species Distribution Models, can provide intel on uncertainty, error quantification and provide probabilities on distribution predictions.

In an era where climate change, overfishing, marine litter, coastal development and other stressors are increasingly threatening marine habitats and biodiversity^2,3,48,49^ there is an increased need for higher resolution and up to date information on marine habitats to enable effective action and management^6,7,15,32,50^. This need has also been driving a search for more efficient tools and methods to monitor key biological aspects and stress indicators, such as habitat integrity, physiography, abiotic conditions and biodiversity. Focused on temperate rocky shores, the present study contributes to that call by producing a blueprint (Figs. 2, 7) on how to better integrate low-cost aerial remote sensing and field data to map biotopes and monitor shallow habitats in general (e.g. seagrass, coral reefs), which can be easily included in local, regional or national monitoring programs, international monitoring networks and assist nations in complying with national and international regulations (e.g. monitor progress towards UN Sustainable Development Goal 14, compliance with EU Directive 2008/56/EC) towards a sustainable management of the marine environment. By integrating local in situ data on species composition and discrete organism assemblages, this approach also contributes to increase the level of detail provided in general habitat type classification systems such as the European Nature Information System (EUNIS) habitat types, the EU Habitat Directive Annex I, and the US Coastal and Marine Ecological Classification Standard (CMECS). Finally, the repeated genesis of maps from temporally re-sampled locations can also improve our capacity to chart the change of coastal biotopes through time, a key deliverable in the monitoring of marine ecosystems.

## Methods

### Aerial survey and orthophoto mosaic construction

For the present study a sheltered bay with an area of approximately 80,000 m^2^, in the southeastern section of Madeira Island (Fig. 1), was selected as a study site to test and demonstrate the integration of aerial imagery in ecological assessments and mapping of submerged coastal habitats. The area, facing south, is fairly protected from prevailing northeast quadrant winds and north-west quadrant wave action^51,52^. The aerial survey of the study site was conducted with the DJI Phantom Vision 2+ quadcopter and matching radio controller. Survey flights were planned and controlled through Pix4d Capture v1.3 for iOS and conducted in automated mode, using a U-pattern, at 100 m above ground with the camera set up at 90º to collect nadir imagery with 80% frontal overlap. Three survey flights, conducted in early light to reduce sunlight backscatter, produced a total of 212 images (with 4384 × 3288 pixels each) covering an estimated area of 10.4 ha. In the ground, GPS coordinates of easily identifiable features were collected with a handheld GPS (Garmin eTrex 10) to serve as Ground Control Points (GCPs). Compiled imagery was processed and analyzed with the photogrammetry software Pix4Dmapper Pro v4.3, employing advanced multi-view imagery and Structure-from-Motion (SfM) algorithms, to construct an ortho-photo mosaic and compute a DSM (Digital Surface Model), a DTM (Digital Terrain Model) and RGB reflectance index^33^. Generated DSM and DTM rasters were used to produce contour layers with 2 m vertical and 1 m horizontal resolutions and a minimum of 200 vertices.

### Optical estimation of depth classes

Depth is a key factor in shaping biotopes and organism assemblages' distribution in coastal waters^12,38,53^, but bathymetry acoustic surveys can be challenging and time consuming, especially in shallow waters^8^. In this study we devised a novel strategy and processing approach (Fig. 7) that provides general depth classes in shallow waters by combining photogrammetry (“Aerial survey and orthophoto mosaic construction” section) and optical bathymetry estimation. The optical based method consists on analysing imagery and relies on the principle of decreased water-leaving radiance with increasing depth^34,35^.

Initially, the submerged area of interest was manually selected in Pix4D Mapper and rasters with logarithmic (ln-) transformed reflectance indexes for RGB bands were generated (ln-Red, ln-Green and ln-Blue). The logarithmic (ln-) transformation has the effect of approximately linearizing reflectance data with respect to depth^35^. Wavebands have different water absorptions and, as depth increases, wavebands with higher absorption (with larger wavelength) decrease faster than those with lower absorption^23,34,35^. Considering the shallow depth range of the study area, rasters for ln-Red index and the ratio of ln-Green to ln-Blue indexes were selected for estimating depth. In parallel, DSM generated contours were smoothed, visually inspected and contextually edited in ArcGIS 10.3.1^54^ to exclude convoluted portions of the contour lines and to match known depths (collected during underwater surveys).

To estimate discrete depth classes throughout the area of interest, we opted to use Object-Based Imagery Analysis (OBIA) and classification using eCognition Essentials (v1.3). Object classification of imagery employs spectral, texture, geometric and topological features as part of the process by relying on the segmentation of the images into homogeneous segments generated by one or more criteria (e.g. scale, shape, compactness). Segmentation was followed by a classification routine based on features (e.g. spectral values, form, size) calculated for each generated segment or object^8,17,55^. Within our approach, compiled rasters were segmented using the multiresolution algorithm over the selected study area (see above; Fig. 2a). Multiresolution segmentation is a bottom-up technique that starts by considering a single pixel as an object and merging it with neighboring ones based on homogeneity criterion^17,56^. Criteria include: a Scale parameter, which affects the size of generated objects; Compactness vs Smoothness ratio, which affects the shape criteria, and; Shape vs Color ratio, which affects whether color or shape has more influence in generating an object. In this study, Scale was set to 65 (eCognition Essentials default setting), while Shape criteria and Shape vs Color ratio were both set to 0.5 to provide equal weight to each criterion. Previously edited DSM contours (produced in Pix4Dm Mapper) were used as reference samples of the logarithmic (ln-) transformed Red index and of the ratio of ln-transformed Green index to ln-transformed Blue index layers (see above; Fig. 2b). A K-Nearest Neighbors (KNN) classifier^57^ was employed to classify segments (i.e. generated objects), which were then merged by category (i.e. depth class) and smoothed (parameter set to 0.25), producing a discrete depth class map of the study area (Fig. 2c). Generated depth classes were visually compared with depth values from georeferenced survey-transects to assess consistency.

### Bottom nature and substrate classification

Similarly to depth, bottom nature and substrate type are key traits in marine habitats that often act as important factors in shaping typical assemblages and biotopes^12,38^. Focused on sessile benthos living on rocky bottoms, a dominant substrate class was established for each underwater survey as "boulders" (mostly with rocks between 15 and 100 cm in diameter), "blocks” (rocks with 100 cm diameter or more) or "platforms" (horizontal or near horizontal rocky "plateau”). Vertical and near vertical surfaces were not considered as they were not included during underwater surveys.

As reflectance of the bottom varies with both depth and substrate type^34,35^, having depth-classes priory established (see above) assisted in the process of categorising substrate type. To that purpose, OBIA was used to individually classify substrate type on each of the different depth classes within the area of interest. This choice assured classification over different sub-areas (within a given depth class) that have lower variation and higher homogeneity in regard to depth-related attenuation of different wavebands, allowing the classification process to be more focused on substrate-related differences in reflectance.

Based on mean reflectance values of Red and Green bands as well as DSM and ln-Red Index and ln-Green to ln-Blue ratio rasters of each sub-area (within a discrete depth-class), segmentation was performed using eCognition multiresolution algorithm with the same criteria settings as previously described (scale: 65; compactness and shape: 0.5; shape and color: 0.5). For each considered depth range, samples were manually selected for different substrate categorical classes (i.e. Platforms, Boulders, Blocks, Sand and not-applicable) followed by the classification of targeted area. Substrate classification was performed with KNN algorithm (k set to 1) using mean values of Normalized Difference Soil, Water and Vegetation Index (NDSI, NDWI and NDVI, respectively, modified by using the Red Index band instead of near infrared) as well as the RGB bands and the DSM raster. Resulting classification shapefiles were then manually inspected, merged by category (substrate) and smoothed (set to 0.25). The procedure was repeated for each of the considered depth categories and resulting shapefiles were compiled and merged into a single file in ArcGIS v10.3.1^54^, with a classification of rocky substrate over the entire extent of the study area. Generated substrate classes were also visually compared with aerial imagery^33^ and with dominant substrate data (from georeferenced survey-transects) to assess consistency and perform contextual editing.

### Benthos surveys

To assess the composition and distribution of conspicuous sessile organism assemblages within the study area, scientific divers conducted underwater surveys over rocky bottoms. Considering the depth-related zonation present in rocky reef^58,59^ and the limitations in assessing depth and bottom nature from aerial imagery^8,29,31,60^, underwater photoquadrat surveys targeting sessile organisms were conducted in two shallow depth ranges: 4–6 m and 9–12 m. During each survey a buoyant GPS logger was deployed and anchored to the bottom for georeferencing the sampling location where six haphazardly placed photoquadrats were collected (in an estimated 5-m radius from the anchoring point). Photographs were taken parallel to the quadrat frame with an Olympus OMD10 equipped with a Panasonic Lumix G 8 mm f/3.5 lens inside a Nauticam NA-ED10 housing and with S&S YS-01 strobes. Additionally, georeferenced 10 m transects (n = 8), with 100 intersection points (one each 10 cm) were used to assess depth, substrate and benthos composition for independent validation of estimated biotope distribution (extrapolated from conspicuous assemblages identified in photo-quadrats, the depth and substrate conditions where they occur and the maps derived from aerial surveys).

### Benthic community assessment

Biota relative abundance and community structure at each sampling site (n = 14) was assessed by analysing all the eighty-four photoquadrats (six photoquadrats for each sample). Each photoquadrat image belonging to the fourteen samples was analysed in Coral Point Count with Excel extensions software (CPCe v4.1^61^) using 100 points distributed in random-stratified fashion (25 points over 4 cells). Intersection points were inspected and labelled with taxonomic categories and subcategories and percent-cover was estimated for each quadrat^58,61^.

All sample data was compiled for further analysis in PRIMER v7 with PERMANOVA+ add on^62^, where relative abundance for each sample (set of six quadrats) was pruned of non-valid and non-living scores (i.e. shadow and frame for the former; sand, rock, rubble for the latter), standardized (i.e. to the sum of all pruned scores) and square-root transformed, for analysis of the sessile community structure^36^. Bray–Curtis similarity matrices were computed and used to determine significantly different grouping and ordination of samples based on a hierarchical clustering and a SIMilarity PROFile routine (SIMPROF)^36,37,63,64^. Non-metric Multidimensional Scaling (nmMDS) plots were produced for visual inspection and to assess potential patterns in assemblage occurrence and distribution. SIMilarity PERcentages analysis (SIMPER) was used to provide detailed information on the composition and taxa contribution towards ordination and grouping resulting from SIMPROF (i.e. organism assemblages)^36,37,58,64^.

Distance based Linear Modelling (DistLM), with a distance based Redundancy Analysis (dbRDA) plot, was used to assess the shaping of community structure (biota relative abundance in each sample and in each quadrat) by depth classes and substrate categories (normalised) and determine matching of identified biological assemblages to unique environmental conditions (i.e. depth and substrate)^36,59,65^. The influence of substrate and depth in the structuring of quadrat data was further confirmed by inspecting the correlation of normalised depth and substrate categories with axes of a Canonical Discriminant Analysis (CDA) designed to best discriminate data grouped by sample-generated SIMPROF grouping (i.e. organism assemblages)^36,65^. Thus, biotopes identified based on conspicuous organism assemblages matching unique depth and substrate conditions were corroborated by DistLM and provide a classification model for extrapolating biotope occurrence over specific depth and substrate conditions^36,59,65^.

The distribution of the four identified biotopes was extrapolated by selecting all discrete areas (i.e. merged segments) in the maps (produced from aerial imagery analysis and classification) where depth and substrate conditions matched those of each of the biotopes and labelling them accordingly (e.g. all segments with Boulders between 3 and 6 m were labelled as Biotope with assemblage c.). For each biotope, new spatial layers were produced and merged to provide a distribution map and estimate their spatial extent (Supplementary Table S1).

Finally, in order to validate the Biotope predicted distribution, point-intersect data of georeferenced transects (independently collected) were labelled based on the biotope predicted to be present on their location. Following, a Bray–Curtis Similarity matrix was computed and an Analysis of Similarity (One-way ANOSIM) used to assess if the predicted grouping was significant in the data ordination.

## Supplementary Information

Supplementary Information.

## Data Availability

The datasets generated and/or analysed during the current study are available from the corresponding author on reasonable request.
