# Supplementary material for: Novel approach to enhance coastal habitat and biotope mapping with drone aerial imagery analysis
Source: Sci Rep. 2021 Jan 12;11:574. doi: 10.1038/s41598-020-80612-7 (PMC7804263; doi:10.1038/s41598-020-80612-7)
Supplement: Supplementary file 1 — Supplementary Information. [file 41598_2020_80612_MOESM1_ESM.docx]

*Novel approach to enhance coastal habitat and biotope mapping with drone aerial imagery analysis*

***João Gama Monteiro^1^**, Jesús L. Jiménez^1^, Francesca Gizzi^1^, Petr Přikryl^1,2^, Jonathan S. Lefcheck^3^, Ricardo S. Santos^4,5^ and João Canning-Clode^1,6^

**1** MARE - Marine and Environmental Sciences Centre, ARDITI - Agência Regional para o Desenvolvimento da Investigação, Tecnologia e Inovação (Funchal, Madeira, Portugal);

**2** University of Ostrava, Faculty of Science, Department of Biology and Ecology (Ostrava, Czech Republic);

**3** Tennenbaum Marine Observatories Network, MarineGEO, Smithsonian Environmental Research Center (Edgewater, Maryland, United States);

**4** Ministry of the Sea, Avenida Doutor Alfredo Magalhães Ramalho, 1465-165 (Algés, Portugal);

**5** University of the Azores, (Horta, Azores, Portugal);

**6** Smithsonian Environmental Research Center (Edgewater, Maryland, United States)


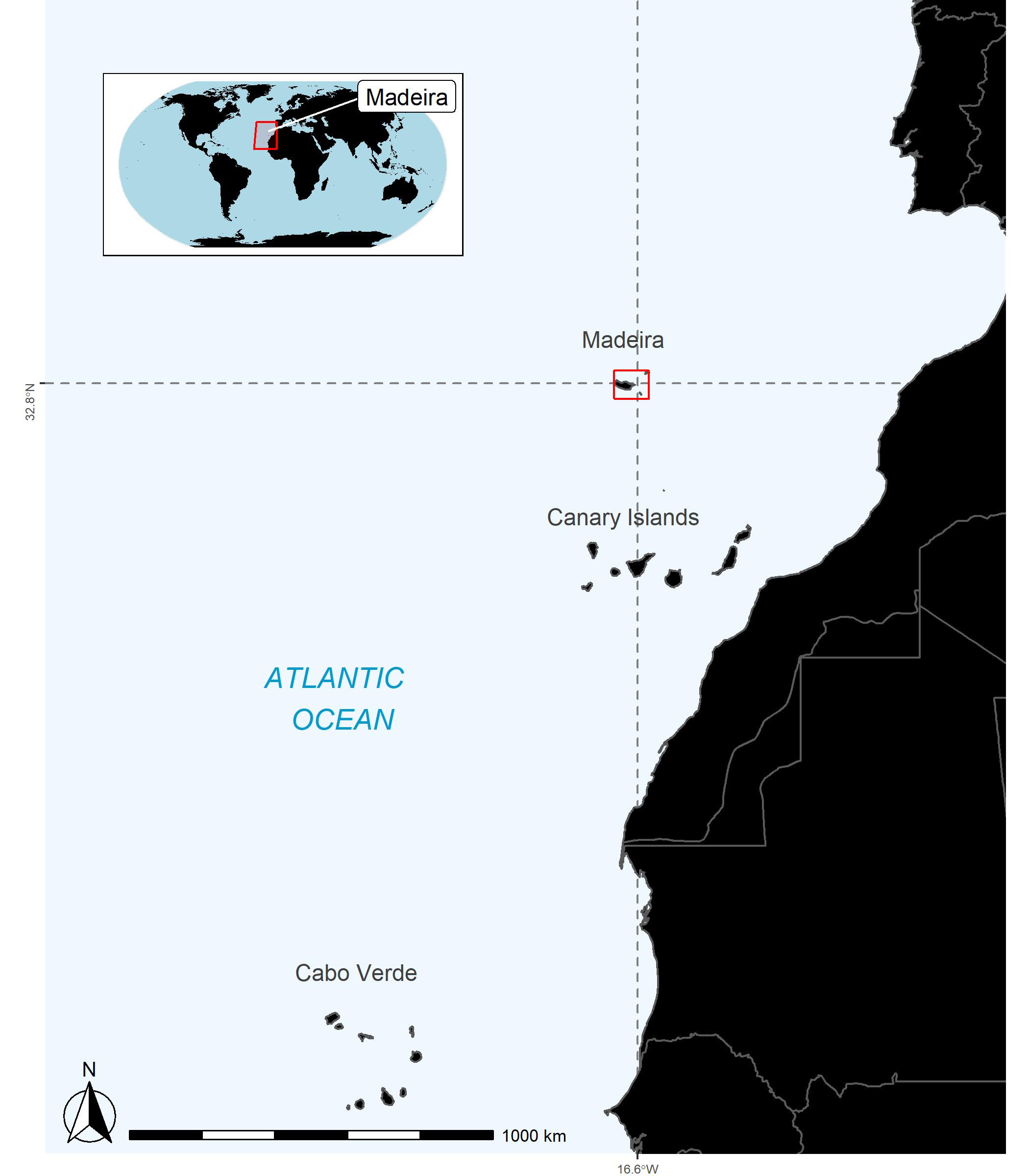


Figure S1: Geographic location of Madeira island in the North-East Atlantic.

Supplementary Table S1: Organism assemblage matching and estimated areas for substrate categories, depth classes and biotopes (a-d).

|  | Depth Classes | | | | |  |
| --- | --- | --- | --- | --- | --- | --- |
| Substrate | **0-3 m** | **4-6 m** | **7-8 m** | **9-11 m** | **11-14 m** | Total |
| **Blocks** | 528.5 m^2^ | 2935.0 m^2^  *Biotope d.* | 1502.4m^2^ | 3175.4 m^2^  *Biotope d.* | 409.3 m^2^ | 85559.7 m^2^ |
| **Boulders** | 2702.7 m^2^ | 6543.0 m2  *Biotope c.* | 1311.6 m^2^ | 1446.0 m^2^  *Biotope b.* | 0 m^2^ | 12003.4 m^2^ |
| **Platforms** | 1868.9 m^2^ | 3917.2 m2  *Biotope a.* | 765.2 m^2^ | 822.4 m^2^ | 31.5 m^2^ | 7405.3 m^2^ |
| **Target area** | 39331.8 m^2^ | | | | | |
| **Submerged** | 79493.95 m^2^ | | | | | |

Supplementary Table S2: Distance-based Linear Modelling summary outputs for predicting Bray-Curtis similarities between photoquadrat samples (n=14 of six quadrats each) with Substrate and Depth class (normalised) as predictors; marginal tests provide details on significant predictors (p<0.05 in bold), best identified solution (in bold) and the relative of variation (%) explained by each DistLM axis.

|  | **Marginal Tests** | | | |
| --- | --- | --- | --- | --- |
| **Predictor** | **SS(trace)** | **Pseudo-F** | **P** | **Prop.** |
| Substrate | 3535.9 | 4.255 | **0.007** | 0.26177 |
| Depth | 3006.6 | 3.4357 | **0.009** | 0.22258 |
|  | **Best Solutions** | | | |
| **Predictor** | **Nº predictors** | **AICc** | **R^2^** | **RSS** |
| Substrate | 1 | 97.05 | 0.26177 | 9971.9 |
| Depth | 1 | 97.774 |  | 10501 |
| Subs.+Depth | ***2*** | ***95.295*** | ***0.4858*** | ***6945.7*** |
|  | **Best Solution** | | | |
|  | **Explained variation (fitted)** | | **Explained variation (total)** | |
| **Axis** | **Individual** | **Cumulative** | **Individual** | **Cumulative** |
| 1 | 54.18 % | 54.18 % | 26.32 % | 26.32 % |
| 2 | 45.82 % | 100 % | 22.26 % | 48.58 % |
